# Eye-Tracking Technology and the Dynamics of Natural Gaze Behavior in Sports: A Systematic Review of 40 Years of Research

**DOI:** 10.3389/fpsyg.2017.01845

**Published:** 2017-10-17

**Authors:** Ralf Kredel, Christian Vater, André Klostermann, Ernst-Joachim Hossner

**Affiliations:** Movement Science, University of Bern, Bern, Switzerland

**Keywords:** eye movements, gaze behavior, visual search, eye-tracking, sports

## Abstract

Reviewing 60 studies on natural gaze behavior in sports, it becomes clear that, over the last 40 years, the use of eye-tracking devices has considerably increased. Specifically, this review reveals the large variance of methods applied, analyses performed, and measures derived within the field. The results of sub-sample analyses suggest that sports-related eye-tracking research strives, on the one hand, for ecologically valid test settings (i.e., viewing conditions and response modes), while on the other, for experimental control along with high measurement accuracy (i.e., controlled test conditions with high-frequency eye-trackers linked to algorithmic analyses). To meet both demands, some promising compromises of methodological solutions have been proposed—in particular, the integration of robust mobile eye-trackers in motion-capture systems. However, as the fundamental trade-off between laboratory and field research cannot be solved by technological means, researchers need to carefully weigh the arguments for one or the other approach by accounting for the respective consequences. Nevertheless, for future research on dynamic gaze behavior in sports, further development of the current mobile eye-tracking methodology seems highly advisable to allow for the acquisition and algorithmic analyses of larger amounts of gaze-data and further, to increase the explanatory power of the derived results.

## Introduction

In sports-related research on motor performance, it has been regularly found that picking up relevant visual information is required to effectively perform a variety of tasks (e.g., Williams et al., 2004). For example, more task efficient—i.e., fewer fixation of longer duration at task-relevant information—and more consistent—i.e., similar gaze pattern over consecutive trials—visual information pick-up is found in expert when compared to novice goal keepers in a penalty-kick-anticipation task (Savelsbergh et al., 2002). Moreover, differences in gaze behavior have also been found on an intra-individual level. For example, Piras and Vickers (2011) reported longer gaze anchoring at a visual pivot during saves than goals in a penalty task for intermediate goal keepers. To measure visual information pick-up in such sport tasks, researchers must assess the participants' gaze behavior either by indirect or by direct methods.

*Indirect methods* are based on the so-called occlusion paradigm that obstructs relevant visual cues, either spatially (i.e., regarding specific areas of interest; e.g., Müller et al., 2006, Experiment 2) or temporally (i.e., over defined time intervals; e.g., Farrow and Abernethy, 2002). The analysis of performance measures together with spatial or temporal occlusions provides valuable insights into the relative importance of occluded visual cues, and further, promotes the derivation of recommendations for optimal gaze behavior. However, these indirect methods have been repeatedly criticized as natural visual information pick-up is considerably restricted (e.g., Farrow and Abernethy, 2003). In response to the occluded information, alternative search strategies may be implemented that might not reflect gaze behavior under unconstrained conditions (e.g., Mann et al., 2007). In contrast, *direct methods* require the application of eye-tracking devices for the direct measurement of eye movements in response to different sensorimotor tasks. Already in 1967, Yarbus presented video-recording techniques to analyse eye movements while participants viewed static pictures and natural scenes (Yarbus, 1967). To an increasing degree over recent decades, mobile eye-tracking devices have been applied as by Savelsbergh et al. (2002). These systems assess the movement of the eye superimposed onto a scene video with a positional cursor representing the current gaze point. Thus, the direct measure does not require the manipulation of the visual stimulus.

The history of sports-related research with eye-tracking devices dates back to 1976, when Bard and Fleury (1976) published a study on gaze behavior in basketball. The participants, five experts and five novices, had to imagine themselves as the ball carrier and to make decisions (i.e., shoot, dribble, pass to a specific player, or none of these actions) in game situations that were schematically presented on slides. Over the decision-making process, the number of fixations as well as their locations, in reference to pre-defined areas of interest, were examined. Most importantly, compared to the novices, the experts were found to show fewer fixations. However, since the eye-tracking device required participants to remain in a seated position and the game situations were presented on static slides, it is questionable whether this finding can be generalized to the highly dynamical situations in real-world sports.

Such issues of external validity of studies on perceptual-cognitive skills were thoroughly discussed by Mann et al. (2007) as well as by Gegenfurtner et al. (2011). Both meta-analyses revealed moderator effects of the stimulus-presentation mode (i.e., the way of presenting the experimental stimuli to the participants), indicating that an increase in the presentation's external validity reveals more pronounced expertise effects in gaze behavior and decision-making. In addition, Gegenfurtner et al. (2011) showed that differences between experts and novices are larger when the task is more controlled by the user and more complex processes are involved.

Together, these meta-analyses suggest that sports-related perceptual-cognitive skills should be examined in naturalistic environments that mimic the complexity of the given task as close as possible. Consequently, applying eye-tracking technology directly in the field generally seems to be the method of choice. However, Mann et al. (2007) advise that, from a scientific perspective, it is also necessary “to pay particular attention to the level of experimental control achieved when testing in the naturalistic environment” (p. 474). Hence, to balance both requirements, pursuing one of two alternative avenues seems advantageous: namely, conducting either (a) field studies, while concurrently paying attention to experimental control, or (b) laboratory studies while concurrently paying attention to external validity.

**Figure 1:**
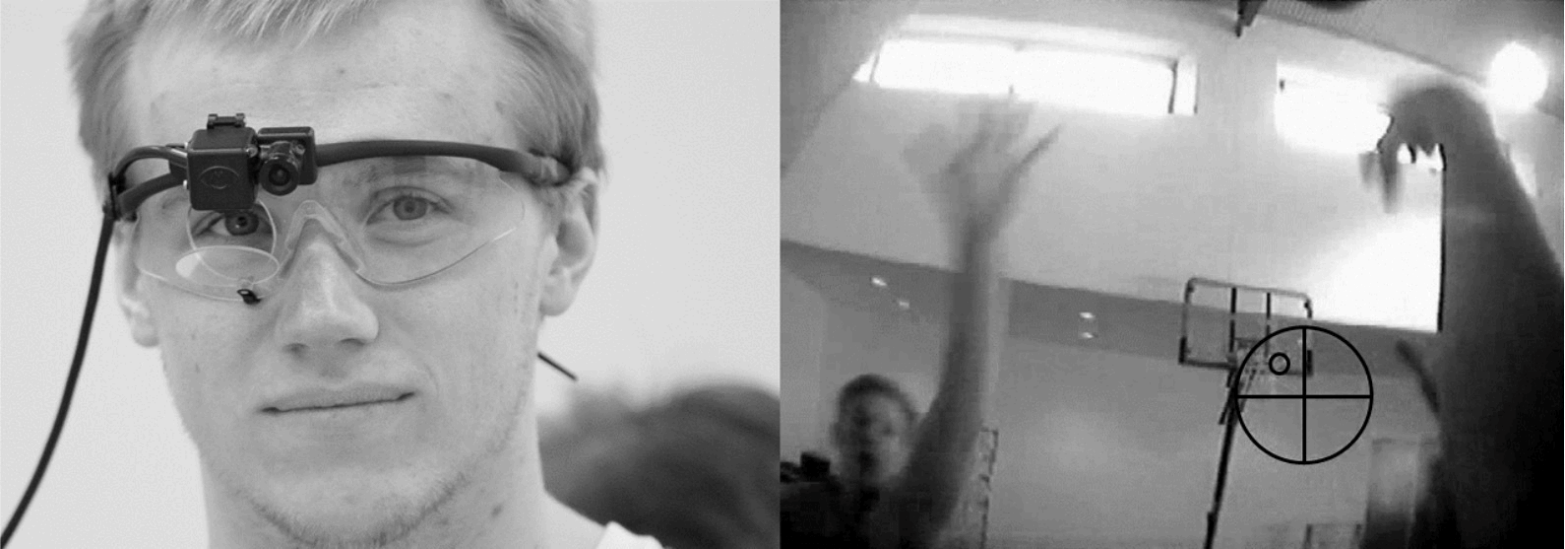
Participant wearing an *Applied Science Laboratories* (ASL; Bedford, MA) mobile eye-tracker (**Left**; monocular set-up with a scene camera and an eye camera capturing eye movements via a mirror in front of the participant's right eye) and an exemplary image captured by the scene camera with the circle indicating the current gaze position (**Right**; the cross hairs denoting the detection of the pupil) (left picture © Ledchumanasarma).

When favoring direct over indirect measurement methods in sports-related research on perceptual-cognitive skill, as has been supported thus far, and moreover, striving for a high external validity of the measurements, the question arises to what degree state-of-the-art mobile eye-tracking technology allows for the reliable acquisition of gaze data in (to the greatest possible extent) unrestricted test conditions. Consequently, a closer first look into the currently available eye-trackers seems worthwhile. As illustrated in Figure 1 (left), the current standard of mobile eye-tracking devices utilizes miniature cameras (with sample rates of 25–60 Hz) mounted on frames, worn like glasses, to capture images of the eye. The capturing is performed either monocularly or binocularly, and directly or indirectly, where the latter applies infrared (IR) reflective mirrors in front of the eyes. To spatially localize gaze in the current field of view, an additional camera (with standard frame rates up to 60 Hz) can be mounted on the glasses' frame to simultaneously capture the scenery in front of the participant (referred to as “scene camera”).

Current customary eye-tracking systems can be categorized into passive-illumination and, more prevalently, active-illumination systems. Unlike the passive systems, the active systems use a combination of IR-LEDs to illuminate the eye and daylight filters to block any radiation other than IR from the camera lens. This method thus assures high-contrast images fairly independent of environmental lighting conditions. In order to process these raw images to extract the current position of the pupil relative to the image captured by the scene camera, both systems require compact computers to be worn on the hip or in a backpack.

To obtain the gaze direction in space, currently available eye-trackers need to be calibrated. During the calibration procedure, the eye movements are gauged in relation to known metric information of a visual pattern presented before the participant, such that rotation angles of the eye ball can be determined in relation to the eye-tracking device. After calibration, the current gaze direction can then be, for instance, mapped onto the scene camera image. This gaze-overlay video highlighting the foveated region (e.g., by a circle; see Figure 1 right) is the standard output of current mobile eye-trackers.

The resulting videos are typically manually analyzed frame-by-frame. The current gaze point is generally allocated to a pre-defined area of interest (AOI; e.g., the opponent's upper body) if either the foveated region overlaps with this area or the gaze is closer to this area than to all other pre-defined AOIs (for recent overviews on further analysis methods, e.g., Duchowski, 2007; Vansteenkiste et al., 2015). From these allocations and—if analyzed—their dynamics over consecutive frames, (object-related) fixations and eye movements can be determined, in turn, allowing for the derivation of further aggregated gaze variables (fixation duration, number of fixations, saccade-related measures, overall viewing time, dynamics of fixations on AOIs, etc.).

However, the mobile eye-tracking devices generally used in sport-science research are far more problematic than those used in fundamental research on gaze behavior. Fundamental studies are generally conducted in a seated position with stationary and technologically sound eye-tracking systems that, among others, feature high-frequency data acquisition and an algorithmic gaze-cue allocation (for a review, see Mele and Federici, 2012). Conversely, in sport-science studies, in addition to the artifacts caused by mobile eye-tracker slips due to head accelerations and the system's inertia, the manual approach of assigning gaze locations to pre-defined AOIs satisfies neither objectivity nor reliability standards. First off, the assignment is more often based on the general conjecture of proximity rather than on any systematic rationale, while secondly, the time-consuming manual procedure considerably limits the total number of analyzed frames per trial, trials per participant, and participants per study (for an in-depth discussion of these issues, see Kredel et al., 2015).

In summary, when aiming to study natural gaze behavior in sports with the direct method of eye-tracking, researchers are confronted with a severe trade-off between two requirements: optimizing the external validity of the experimental conditions and achieving sufficient objectivity and reliability of the measurements. Hence, a valuable closer look is taken to consider how this issue has been treated by researchers over the last 40 years, dating back to 1976 when the very first sport-related eye-tracking study was published by Bard and Fleury (1976). To this end, a systematic review was conducted to characterize the publications, the researched tasks, the applied eye-tracking devices and analyses methods as well as the derived gaze measures in sports-related eye tracking.

## Methods

**Figure 2:**
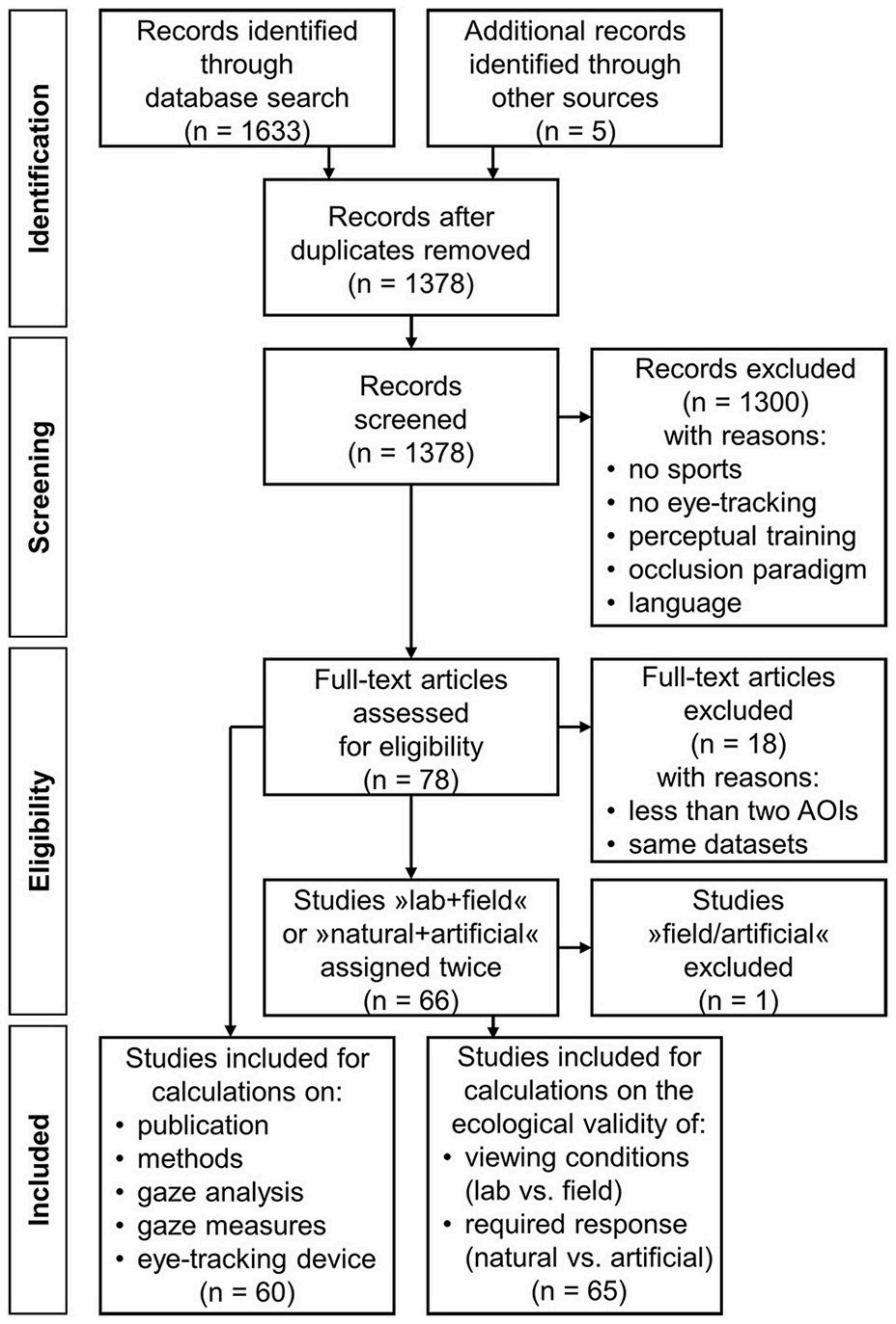
Flow diagram depicting the selection of relevant literature from identification to final inclusion of sports-related eye-tracking studies on the dynamics of natural gaze behavior (1976-2016) (PRISMA 2009 flow diagram, adapted from Shamseer et al., 2015).

This systematic review was conducted in lines with the PRISMA guidelines (*P*referred *R*eporting *I*tems for *S*ystematic reviews and *M*eta-*A*nalyses; Shamseer et al., 2015). Figure 2 illustrates the whole process as a PRISMA flow diagram, from the initial stage of study identification to the final stage of study inclusion.

### Literature search

The following electronic databases were used: Web of Science, PubMed Central, SPORTDiscus, and ScienceDirect. For the literature search in each of these databases, the keyword *sport* was combined with each of the following keywords: eye tracking, gaze behavio^*^r, eye movement, and *visual search*. Further studies were identified as cited references in relevant articles, personal collections, and cross-references.

### Inclusion and exclusion criteria

Studies were included if (a) they were written in the English language, (b) they were published in peer-reviewed journals, and (c) natural, dynamic visual behavior was examined, meaning that gaze was assigned to at least two different AOIs. Consequently, for instance, studies on the so-called “Quiet Eye” phenomenon (cf. Vickers, 2007) were excluded to a large extent, as these studies regularly assess only one fixation location. Furthermore, studies without eye-tracking, perceptual-training studies, and studies exclusively based on the temporal or spatial occlusion paradigm were excluded.

### Identification, screening, and eligibility

In total, 1633 studies were identified using the literature-search criteria specified above (in some cases, stemming from publications in which more than one experiment is presented, as is the case, for instance, in the two studies reported by Abernethy, 1990). After removing duplicates, the abstracts of 1,378 studies (now including an additional five studies identified from other sources) were screened by two independent raters and omitted from the review if: there was no sports objective, no eye-tracking data was acquired, the study focused on perceptual-training effects, an occlusion paradigm was applied, or the manuscript was not written in English language. In cases of opposing screenings, the respective papers were screened again by both raters and discussed until a final decision was reached. Over the screening process, 1,300 studies were excluded. In the eligibility phase, the remaining 78 papers were screened in entirety by the two raters. At this stage, 18 further studies were excluded because either less than two AOIs were analyzed, thus not allowing for the derivation of statements on dynamic visual behavior, or they only aimed to re-analyse previously published data sets. The remaining 60 papers, listed in Table 1, were used for analyses of specifics of the publication and details of the researched tasks, gaze-analysis procedures, and derived gaze measures.

**Table 1:** Overview of sports-related eye-tracking studies (1976–2016; for an explanation of the descriptors of the publication, task, gaze analysis, and gaze measures, see Table 2).

**Publication**	**Task**					**Gaze analysis**				**Gaze measures**					
**Author(s)/ Year**	**Sport**	***N***	**Condition**	**View**	**Response**	**Trials**	**ET**	**GCA**	**NAOI**	**FD**	**NF**	**SA**	**VT**	**DN**	**DE**
Bard and Fleury, 1976	Basketball	10/10	Lab	Bird	Artificial	–/84	m/–	Manual	12	–	x	–	x	–	–
Abernethy and Russell, 1987	Badminton	31/31	Lab	1st	Artificial	320/320	m/25	Manual	7	x	x	–	x	x	–
Goulet et al., 1989	Tennis	29/29	Lab	–	Artificial	–/54	m/30	Manual	11	x	x	–	–	x	–
Ripoll, 1989	Table Tennis	5/5	Field	1st	Natural	20/60	m/–	Manual	3	x	x	–	–	x	–
Abernethy, 1990 (Exp1)	Squash	32/32	Lab	1st	Artificial	160/160	m/–	Manual	9	x	x	–	x	x	–
Abernethy, 1990 (Exp2)	Squash	8/8	Field	1st	Artificial	80/80	m/–	Manual	9	x	x	–	x	x	–
Vickers, 1992	Golf	12/12	Field	1st	Natural	20/20	m/30	Manual	3	x	x	x	x	–	x
Ripoll et al., 1995	Boxing	18/18	Lab	1st	Artificial	22/22	m/30	Manual	6	x	x	–	x	x	–
Vickers, 1996	Basketball	16/16	Field	1st	Natural	20/20	m/30	Manual	6	x	x	x	x	–	x
Vickers and Adolphe, 1997	Volleyball	12/12	Field	1st	natural	20/20	m/30	Manual	7	x	–	x	x	–	x
Williams and Davids, 1997	Soccer	20/20	Lab	3rd	Artificial	22/22	m/50	Manual	3	–	x	–	x	x	–
Singer et al., 1998	Tennis	5/6	Field	1st	Natural	10/10	m/50	Manual	4	x	x	x	–	–	x
Williams and Davids, 1998	Soccer	24/24	Lab	1st	Natural	18/18	m/50	Manual	4	x	x	–	x	x	–
Helsen and Starkes, 1999 (Exp2)	Soccer	28/28	Lab	1st	Artificial	30/30	m/50	manual	4	x	x	–	x	–	–
Helsen and Starkes, 1999 (Exp3)	Soccer	28/28	Lab	1st	Natural	30/30	m/50	Manual	7	x	x	–	x	–	–
Williams and Elliott, 1999	Karate	16/16	Lab	1st	Natural	30/30	m/50	Manual	6	x	x	–	x	x	–
Kato and Fukuda, 2002	Baseball	18/18	Lab	1st	Artificial	10/10	s/30	Manual	8	x	x	–	x	–	x
Savelsbergh et al., 2002	Soccer	14/14	Lab	1st	artificial	30/30	m/50	Manual	9	x	x	–	x	–	x
Ward et al., 2002	Tennis	16/16	Lab	1st	Natural	32/32	m/30	Manual	7	x	x	–	x	x	–
Williams et al., 2002a	Billiard	24/24	Field	1st	Natural	30/60	m/30	Manual	5	x	x	–	x	–	–
Williams et al., 2002b	Table Tennis	6/10	Field	1st	Natural	6/6	m/60	Manual	4	x	x	–	x	–	–
Williams et al., 2002c	Tennis	16/16	Lab	1st	Natural	16/16	m/–	Manual	7	x	x	–	x	x	–
Martell and Vickers, 2004	Ice Hockey	12/12	Field	1st	Natural	8/8	m/30	Manual	6	x	x	x	x	–	x
McPherson and Vickers, 2004	Volleyball	5/5	Field	1st	Natural	12/35	m/30	Manual	5	x	x	x	x	–	x
Nagano et al., 2004	Soccer	8/8	Field	1st	Natural	9/9	m/30	Manual	7	–	–	–	x	–	x
Savelsbergh et al., 2005	Soccer	16/16	Lab	1st	Artificial	30/30	m/50	Manual	4	x	x	–	x	–	–
Nagano et al., 2006	Soccer	8/8	Field	1st	Natural	9/9	m/30	Manual	7	x	x	–	x	–	x
Panchuk and Vickers, 2006	Ice Hockey	8/8	Field	1st	Natural	40/40	m/30	Manual	8	x	x	x	x	–	–
Vickers, 2006	Skating	5/5	Field	1st	Natural	1 round	m/30	Manual	4	x	–	–	x	–	–
Vaeyens et al., 2007	Soccer	40/65	Lab	3rd	Natural	33/33	m/60	Manual	9	x	x	–	x	x	–
Nieuwenhuys et al., 2008	Climbing	12/12	Field	1st	Natural	2 routes	m/50	Manual	4	x	x	–	x	–	–
North et al., 2009	Soccer	18/26	Lab	3rd	Artificial	48/48	m/50	Manual	5	x	x	–	x	x	–
Takeuchi and Inomata, 2009	Baseball	14/14	Lab	1st	Nat.+artificial	10/10	m/50	Manual	4	x	x	–	x	–	x
Wilson et al., 2009	Soccer	14/14	Field	1st	Natural	14/14	m/25	Manual	2	x	x	–	–	–	–
Dicks et al., 2010	Soccer	8/8	Lab+field	1st	Nat.+artificial	75/75	m/25	Manual	10	x	x	x	x	–	x
Hagemann et al., 2010	Fencing	21/62	Lab	1st	Artificial	45/45	s/500	Manual	11	x	x	–	x	–	–
Piras et al., 2010	Volleyball	30/30	Lab	1st	Artificial	120/120	s/500	Manual	7	x	x	x	x	x	–
Savelsbergh et al., 2010	Soccer	20/20	Lab	1st	Natural	15/30	m/50	Manual	6	–	–	–	x	–	–
Button et al., 2011	Soccer	8/8	Lab	1st	Nat.+artificial	45/60	m/25	Manual	9	x	x	–	–	–	x
Roca et al., 2011	Soccer	20/20	Lab	1st	Artificial	20/20	m/25	Manual	5	x	x	–	x	x	–
Afonso et al., 2012	Volleyball	27/27	Field	1st	Natural	6/6	m/30	Manual	10	x	x	–	x	–	–
Breslin et al., 2009	Cricket	32/32	Lab	3rd	Natural	10/10	m/25	Manual	5	x	x	–	x	–	–
Roca et al., 2013	Soccer	24/24	Lab	1st	Natural	6/20	m/25	Manual	5	x	x	–	x	–	–
Ryu et al., 2013	Basketball	22/22	Lab	3rd	Artificial	48/48	s/250	Algo.	10	x	x	x	x	x	–
Schorer et al., 2013	Volleyball	40/40	Lab	1st	Artificial	72/72	s/500	Algo.	heat m.	x	x	–	x	–	–
Afonso et al., 2014	Volleyball	9/9	Lab+field	1st	Nat.+artificial	12/12	m/30	Manual	10	x	x	–	x	–	–
Hall et al., 2014	Horse Riding	10/10	Field	1st	Natural	15/15	m/60	Manual	3	x	x	–	x	–	–
Hüttermann et al., 2014	Soccer	22/22	Field	1st	Natural	30/30	m/30	Manual	3	–	–	x	–	x	–
Piras et al., 2014a	Volleyball	30/30	Lab	1st	Artificial	120/120	s/500	Algo.	7	x	x	x	x	x	–
Piras et al., 2014b	Judo	20/20	Field	1st	Natural	40/40	m/30	Manual	7	x	x	x	x	x	–
Ryu et al., 2014	Basketball	38/38	Lab	3rd	Artificial	72/72	s/250	Algo.	10	x	x	x	x	–	–
Timmis et al., 2014	Soccer	12/12	Field	1st	Natural	8/8	m/30	Manual	5	x	x	–	x	–	–
Vansteenkiste et al., 2014a	Volleyball	37/37	Lab	3rd	Natural	20/20	m/60	Manual	5	–	–	–	x	–	x
Vansteenkiste et al., 2014b	Cycling	17/25	Field	1st	Natural	3 tracks	m/25	Manual	9	x	–	–	x	–	–
Vansteenkiste et al., 2014c	Cycling	5/10	Field	1st	Natural	4 km route	m/25	Manual	5	–	–	–	x	–	–
Gorman et al., 2015	Basketball	20/32	Lab	Bird	Artificial	8/12	m/25	Manual	13	x	x	–	x	–	–
Lex et al., 2015	Soccer	20/20	Lab	Bird	Artificial	36/36	s/500	Algo.	Heat m.	x	x	–	x	–	–
Manzanares et al., 2015	Sailing	20/20	Lab	1st	Natural	1 regatta	m/30	Manual	16	x	–	x	–	x	–
Milazzo et al., 2016	Karate	28/28	Field	1st	Natural	21/21	m/30	Manual	17	x	x	–	x	–	–
Murray and Hunfalvay, 2016	Tennis	43/43	Lab	1st	Artificial	18/18	s/60	Manual	3	x	x	–	x	–	–

*A dash indicates that the respective information is not reported in the paper or that the respective gaze measure was not calculated in the study. Studies with an algorithmic approach to gaze analysis are highlighted in gray*.

### Data extraction and quantitative analyses

For a precise characterization of the included studies, four groups of descriptors were defined and are further explained in Table 2. To specify beyond the information presented in Tables 1, 2, we grouped the studies in regards to their publication organ, thereby distinguishing sport-scientific vs. psychological journals and further sub-categorizing with respect to the disciplinary focus. In two cases in which the publication did not clearly provide crucial information on the gaze analysis (i.e., Hagemann et al., 2010; Piras et al., 2010), we contacted the main author of the study.

**Table 2:** Descriptors used in Table 1 for the characterisation of the included studies.

**Category**	**Attribute**	**Explanation**	**Example**
Publication	Year	Year of publication	1999
	Author(s)	Authors of the publication (abbreviated in cases of more than 2 authors)	Ripoll
Task	Sport	Researched kind of sport	Basketball
	*N*	Number of participants for whom gaze behavior was analyzed divided by total number of participants	20/20
	Condition	Visualization conditions: lab (slides or videos) vs. field (*in-situ* investigations)	Lab+field
	View	Viewing perspective: 1st (agent's perspective) vs. 3rd (agent presented on the field) vs. bird (from above)	1st
	Response	Required motor response: natural (natural action) vs. artificial (verbal, button press, joystick, no response)	Natural
Gaze analysis	Trials	Number of trials in which the gaze behavior was analyzed divided by the total number of trials	22/26
	ET	Eye tracker; type: m (mobile) vs. s (stationary); and sampling rate (in Hz)	m/25
	GCA	Gaze-cue allocation: manual (ratings) vs. algo. (algorithmic)	Manual
	NAOI	Number of pre-defined areas of interest gaze could be allocated to (alternatively: heat map illustration)	5
Gaze measures	FD	Fixation durations; x (measured, e.g., as mean fixation duration) vs. – (not measured)	x
	NF	Number of fixations: x (measured, e.g., as average count over a single trial) vs. – (not measured)	–
	SA	Saccades: x (measured; e.g., as average count over a single trial) vs. – (not measured)	x
	VT	Viewing times: x (analyzed, e.g., as sum of fixation durations over a single trial) vs.—(not analyzed)	–
	DN	Description of the dynamics of the fixation behavior that was not related to a specific event: x (analyzed, e.g., as order of fixations) vs.—(not analyzed)	x
	DE	Description of the dynamics of the fixation behavior that was related to a specific event: x (analyzed, e.g., in relation to a particular stimulus) vs.—(not analyzed)	–

The extracted data was described in terms of percentage (%), average (M and Mdn) or extreme (min and max) values, aggregated either over all studies or, if appropriate, over sub-samples of studies, such as certain years of publication or in reference to specific variables of interest. In this latter regard, the variables “condition” and “response” seemed to be particularly relevant. The respective values “field” (rather than “lab”) and “natural” (rather than “artificial”) strongly suggest the study's high external validity, as previously discussed, an important criterion for research on natural gaze behavior. For this reason, the studies listed in Table 1 were additionally grouped into the sub-samples “lab/natural,” “lab/artificial,” “field/natural,” and “field/artificial.” Because two of the 60 studies comprised both lab and field tests with natural as well as artificial responses and two further studies used a lab setting that required both natural and artificial responses, these studies were counted in each sub-sample. Beyond, the only study with a “field/artificial” combination was excluded for statistical reasons, specifically for the calculation of quantitative comparisons regarding the studies' ecological validity. Ultimately, a total of 65 studies were included (see final boxes of Figure 2).

## Results

Below, the studies listed in Table 1 will first be described according to the specifics of the publications, the researched task, the applied gaze analysis (including eye-tracking devices), and the derived gaze measures. Subsequently, relevant sub-samples of studies will be compared, namely, studies with vs. without externally valid viewing and response conditions and studies based on the application of mobile vs. stationary eye-trackers. Relevant trends over time will additionally be reported.

### Publications

Since 1976, 60 studies have investigated natural gaze behavior in sports with eye-tracking technology. About 60% (35) have been published in sport-scientific journals, while the remaining roughly 40% (25) in psychological journals. The papers published in sport-scientific journals are almost equally distributed over journals of four specific perspectives: sport-psychology journals (7; e.g., *Journal of Sport* & *Exercise Psychology*), journals with a focus on motor behavior (8; e.g., *Human Movement Science*), journals with an applied approach (11; e.g., *Research Quarterly for Exercise* & *Sport*), and journals with a multi-disciplinary perspective (9; e.g., *Journal of Sports Sciences*). Similarly, the papers published in psychological journals can also be about evenly distributed over four sub-domains: cognitive psychology (8; e.g., *Cognitive Processing*), perception psychology (7, e.g., *Perception*), applied psychology (5, e.g., *Applied Cognitive Psychology*), and multidisciplinary journals (5, e.g., *Plos ONE*). The fact that more than half of the studies (34) were published over the last 10 years proves that the field of gaze-related research has been gaining considerably increasing importance.

### Task

#### Sport

In terms of the sports researched, tasks from 21 different sports have been investigated thus far ranging from ice hockey and squash to golf and fencing, sailing and even horse riding. As expected, the vast majority of the studies focus on sports in which perception is not only required for movement control but also for decision-making. Thus, 81.7% of the studies apply to sport games, in reference to the “Teaching Games for Understanding” (TGFU) classification proposed by Butler et al. (2003), mainly to invasion games (26; e.g., football), net and wall games (18; e.g., volleyball), or target and striking/fielding games (5; e.g., baseball). The remaining studies regard either combat sports (5; e.g., karate) or sports that require locomotion (6; e.g., cycling).

#### Number of participants (N)

In the studies, on average, 20.6 participants were examined (*min* = 5, *max* = 65); however, in some cases (6), gaze behavior was measured only for a fraction (*min* = 33.9%, *max* = 83.8%) of the whole sample. This reduction was due to either technical issues (e.g., North et al., 2009) or the need to decrease the demands of analysis (e.g., Hagemann et al., 2006). In sum, the gaze data reported so far is based on measurements of substantially more than 1,000 participants (*N* = 1,131).

#### Condition

Gaze data was acquired in natural viewing conditions of the field in 39.4% of cases (24), while 60.6% of the studies had been conducted in an experimenter-controlled lab environment with slide or video presentations. Only in two studies, participants were tested under both viewing conditions. Interestingly, considering 5-year intervals from 1986 to 2015, the percentage of studies conducted in field conditions remained rather constant over the last decades (between 35% and 50% without any trend).

#### View

In the vast majority of studies (49; 83.1%), a first-person viewing perspective was implemented, meaning that the scenery was presented to the participant as if he/she was part of the evolving situation (as is always the case in field conditions, but not necessarily so in lab conditions). In about 10% of the studies (7; 11.9%), all of which researched team games, slides or videos captured from behind the field were displayed including the decisive agent. The participants were then asked to put themselves in the position of this exact player. In the remaining studies (3; 5.1%), the same approach was used, however, with slides or videos captured from a bird's eye view. Increasingly since about the year 2000, researchers have presented stimuli on life-sized screens (*N* = 27 studies) rather than smaller displays (*N* = 10 studies).

#### Response

In slightly more than the half of the studies (34; 60.7%), the participants were asked to respond naturally to the presented situations; for instance, in a field research setting, actually performing a defensive movement in response to an opponent's attack or, in a laboratory setting, at least mimicking a whole-body dynamic response. In the remaining cases (“artificial”: 22; 39.3%), either a spatially reduced motor response (i.e., a button press or a joystick movement) or a verbal response was required. Only, in four studies (i.e., 6.7%), both response modes were used. Considering the developments over the last decades, there has been a distinct increase in studies applying natural response modes, with considerably small percentages in the years before 1996 (25.0%), a steep increase to 2005 (72.2%), and a plateau thereafter (58.8%).

### Gaze analysis

#### Trials

In the included studies, each participant had to perform on average 41.5 trials (*SD* = 49.1, *min* = 6, *max* = 320), from which gaze behavior was analyzed in an average of 37.8 trials (*SD* = 49.9, *min* = 6, *max* = 320). Aside from economic reasons, the reduction of gaze-analysis trials can also be attributed to the research strategy of comparing only extreme cases (e.g., Savelsbergh et al., 2010; Roca et al., 2013). For descriptive purposes, when extreme positive outliers incline one to favor rather the median over the mean as a measure for central tendencies, a typical study on natural gaze behavior in sport can be characterized by 27 trials per participant from which gaze measures were calculated for 20–21 trials.

#### Eye-tracker (ET)

In the vast majority of studies, a mobile eye-tracker (51; 85.0%) rather than a stationary device (9; 15.0%) was used. Thus far, the typical eye-tracker of sports-related research on natural gaze behavior, has a median sample rate of 30 Hz. Quite surprisingly, over the last decades, there has been no remarkable increase of the “standard” operating-frequency (cf. studies later than 2010: *Mdn* = 30 Hz). Nevertheless, it should also be noted that, in recent years, a desire for higher-frequency devices has become apparent, as from 2010 onwards, about two sports-related research studies per year have utilized eye-trackers with a sample rate of 250 Hz or even 500 Hz.

#### Gaze-cue allocation (GCA)

In order to increase the objectivity of the eye-movement data processing, computerizing the analyses has been suggested in recent literature (e.g., Piras et al., 2010; see also Hagemann et al., 2010; Vansteenkiste et al., 2015 proposed a manual fixation-by-fixation gaze-cue allocation method). In Table 1, however, studies were only categorized as “algorithmic” if the gaze allocation to a certain AOI was completely computerized. This approach requires that for a certain point in time, two types of information can be related within the same frame of reference; namely, the current gaze point and the current position of a certain AOI (or current positions of a number of AOIs). This requirement could be met by projecting videos in which the center of an AOI has been digitized in advance, calculating the intersection point between the gaze vector and the screen and allocating gaze to this AOI if the Euclidean distance between AOI and gaze falls below a predefined value. Such an algorithmic approach was pursued in only 8.3% of the studies included in this review (5), whilst in the vast majority of cases the gaze recordings were analyzed manually frame-by-frame as sketched in the introduction (60; 91.7%).

#### Number of AOIs (NAOI)

Rather unchanged since the very first studies, the current gaze point was allocated to about seven AOIs that had been pre-defined by the researchers (*M* = 6.8, *SD* = 3.1, *min* = 2, *max* = 17).

### Gaze measures

**Figure 3:**
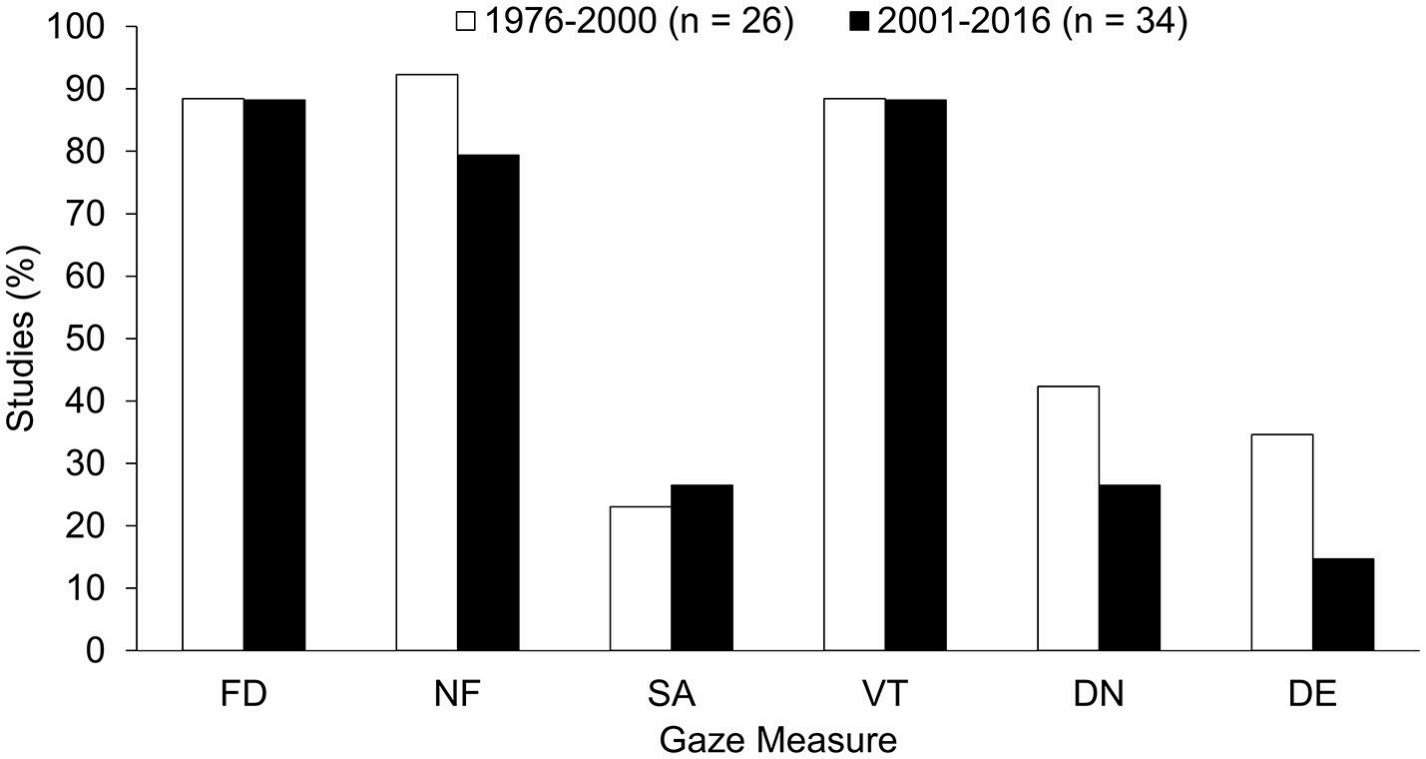
Percentage of studies in the years 1976-2000 and 2001-2016, respectively, in which the following gaze measures were analyzed: fixation duration (FD), number of fixations (NF), saccades (SA), viewing times (VT), non-event-related fixation dynamics (DN), and event-related fixation dynamics (DE).

Figure 3 depicts the percentages of studies in which the gaze measures specified in Table 2 are reported. To allow for the identification of trends, the studies are subdivided into those published up to 2005 (26) and those published in 2006 or later (34). As can be inferred from the figure, two groups of gaze variables can be distinguished from one another. On the one hand, one finds measures that had been calculated in more than 80% of the studies (with no considerable trends over time). All of these variables refer to fixations, namely fixation duration (FD), number of fixations (NF) and viewing time (VT, additively derived from the FD measure). Yet, these measures do not try to order the fixations to sequences or to relate them to certain events. On the other hand, the remaining measures had been calculated in considerably less than 50% of the studies, again without any significant changes over time. These variables concern either saccade-related measures (SA)—which can hardly be acquired with the currently available hardware—or measures of gaze dynamics, concerning the temporal order of gaze behaviors in a non-event-related manner (DN; e.g., order of fixations) or with respect to certain events (DN; e.g., the appearance of a cue in the environment or the initiation of one's own motor response).

### Externally valid test conditions

Two variables listed in Table 1 (and Table 2) are of particular interest to examine the external validity of data-acquisition conditions–namely, the viewing conditions as well as the required motor response, with the values “field” and “natural” implying larger degrees of external validity than values of “lab” and “artificial,” respectively.

Although conceptually clearly distinguishable, on an empirical level, these two variables are mutually dependent. All in all, it would be difficult to find good reason to investigate gaze behavior under *in situ* conditions while demanding non-natural responses from the participants. Indeed, this expectation is confirmed by the fact that the category of “field/other” studies is consequently almost empty. The only respective entry was a study conducted by Abernethy (1990, Exp. 2), who required participants in an *in-situ* task to verbally indicate the perceived direction and the force of opponents' squash strokes. With only this singular case, the “field/artificial” category was neglected in further analyses.

However, since a laboratory setting allows for the investigation of both natural and artificial responses, the remaining three categories were retained, resulting in a total of 65 further considered studies (with studies that applied more than one viewing or response condition counted twice in the following analyses). Of these studies, less than 40% were conducted under field viewing and natural response conditions (25). Only about one-third of the remaining 40 laboratory studies required a natural response (15; 37.5%), while two-thirds an artificial response (25; 62.5%). This bias toward non-natural responses illustrates that the laboratory studies were likely designed to focus on the findings' internal validity in the context at hand; rather focusing on the precise measurement of the participant's response while accepting potential task alterations.

**Figure 4:**
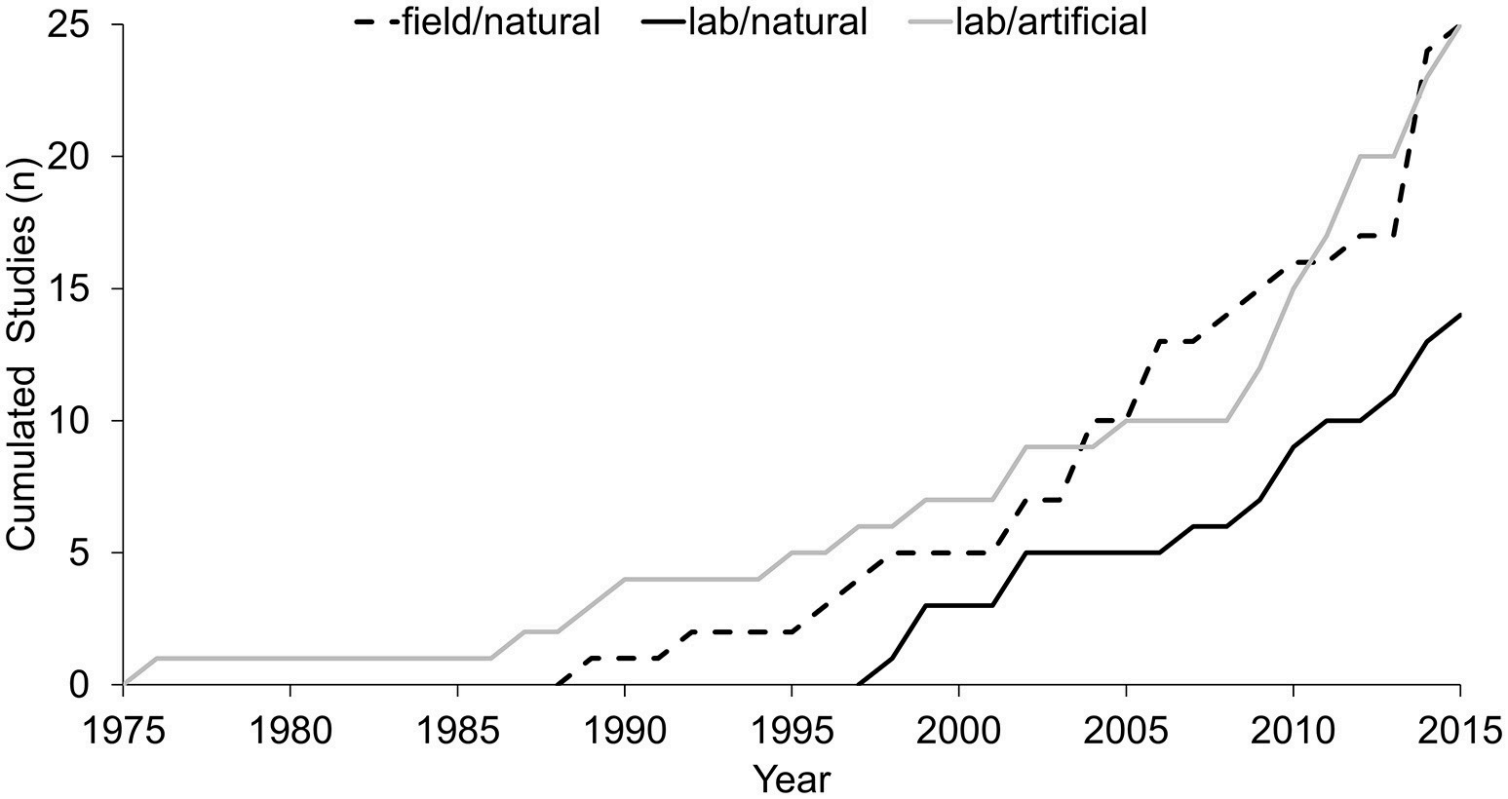
Cumulated number of studies 1976-2015, assigned by the external validity of the viewing conditions (field vs. lab) and the required response mode (natural vs. artificial), respectively to the categories “field/natural,” “lab/natural,” and “lab/artificial.”

Figure 4 depicts the accumulated number of studies in each of the three test-condition categories from 1975 to 2015. As depicted in the figure, laboratory studies with artificial responses were first in the early years of sports-related eye-tracking research. The commencement of field studies started in the 1990s, with increasing development over the last 25 years. Laboratory studies with natural motor responses have been conducted since 1998; however, as these studies show only a moderate increase in recent years, the accumulated number has not yet caught up to that of the other two test-condition categories.

**Figure 5:**
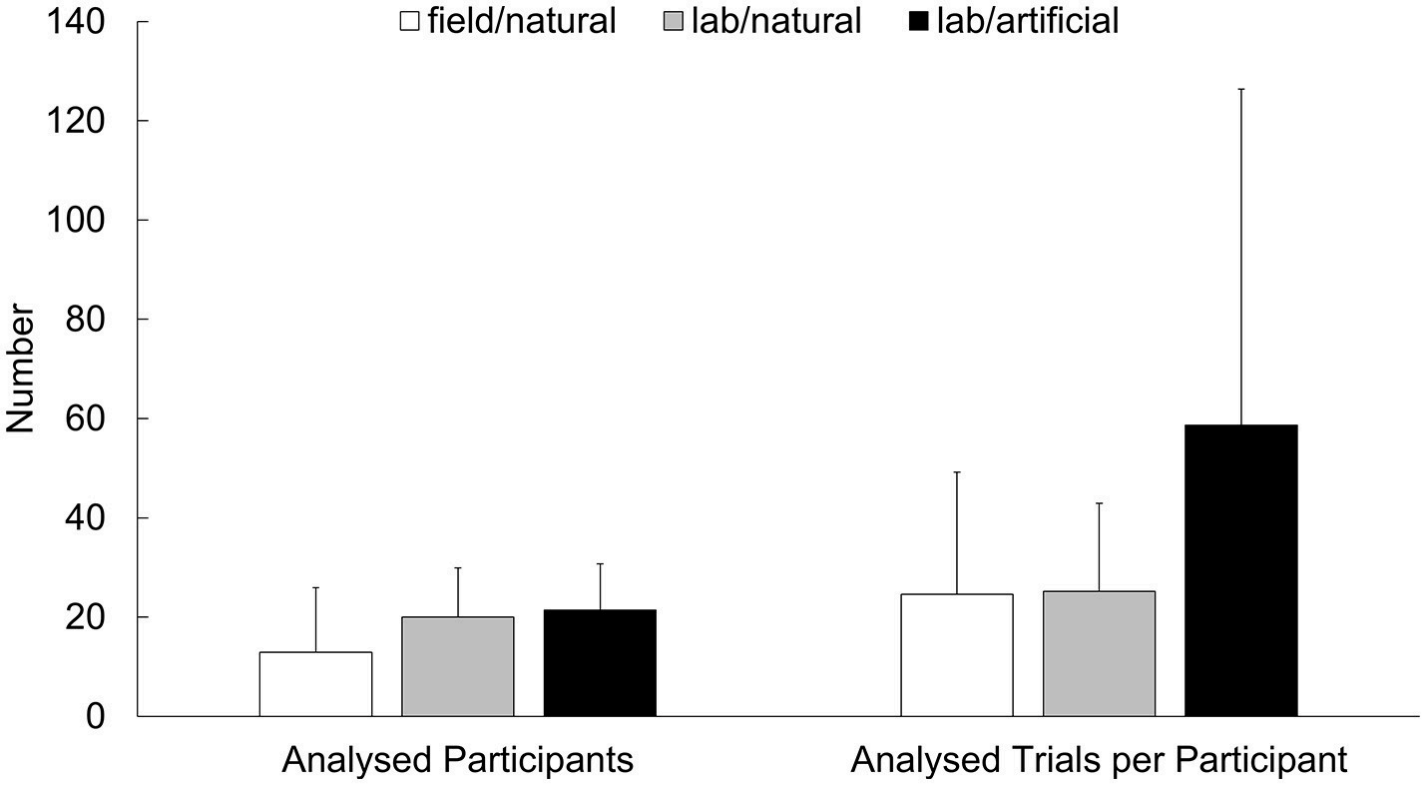
Average numbers of analyzed participants and analyzed trials per participant for the studies in the categories “field/natural,” “lab/natural,” and “lab/artificial.” The error bars denote standard deviation.

When questioning why “lab/artificial” studies are still so prominent in sports-related research on the dynamics of gaze behavior, the major advantages of a laboratory setting should be weighed. Especially, the use of currently available eye-tracking technology is significantly facilitated under standardized viewing conditions and with non-dynamic responses from the participants. As illustrated in Figure 5, the “lab/artificial” studies claim the additional advantages of fostering the highest number of analyzed participants per study and the highest number of analyzed trials per participants. Furthermore, an algorithmic approach to gaze-cue allocation was pursued in—at least—about every fifth “lab/artificial study” (5/26; 19.2%), while no similar approach was taken in any of the “lab/natural” and “field/natural” studies (0/39; 0.0%). However, these benefits are accompanied by the flaw that about one third of the “lab/artificial” studies (9/26; 34.6%) used a stationary eye-tracker, which prevented natural body and head movements as the participants were seated in front of a screen. In contrast, mobile devices were used in all of the “lab/natural” and “field/natural” studies (39/39; 100.0%).

### Mobile eye-tracking devices

When creating sub-samples of studies based on the type of applied eye-tracker, the picture sketched above for the external validity of the test conditions is somewhat mirrored, as the use of a stationary device requires a laboratory environment and non-dynamic responses. Consequently, all of the studies in which stationary eye-trackers were used are “lab/artificial” studies (9/9; 100.0%). From the studies in which mobile devices were applied, about every second was conducted under field conditions (26/51; 51.0%) and in about three quarters of the cases, a naturalistic dynamic response was required from the participants (39/51; 76.5%).

However, it should also be noted that using a stationary eye-tracker comes with a number of advantages. In particular, the devices in stationary eye-tracker studies had considerably higher sampling rates (*M* = 343.3 Hz, *SD* = 188.1 Hz, *min* = 30 Hz, *max* = 500 Hz) compared to those in mobile eye-tracker studies (*M* = 36.7 Hz, *SD* = 11.9 Hz, *min* = 25 Hz, *max* = 60 Hz). Furthermore, the application of stationary devices allowed for greater numbers of analyzed participants per study (*M* = 27.3, *SD* = 8.4, *min* = 18, *max* = 43; mobile eye-tracker studies: *M* = 17.1, *SD* = 8.8, *min* = 5, *max* = 40) and of analyzed trials per participant (*M* = 57.4, *SD* = 37.3, *min* = 10, *max* = 120; mobile eye-tracker studies: *M* = 33.4, *SD* = 51.4, *min* = 3, *max* = 320).

**Figure 6:**
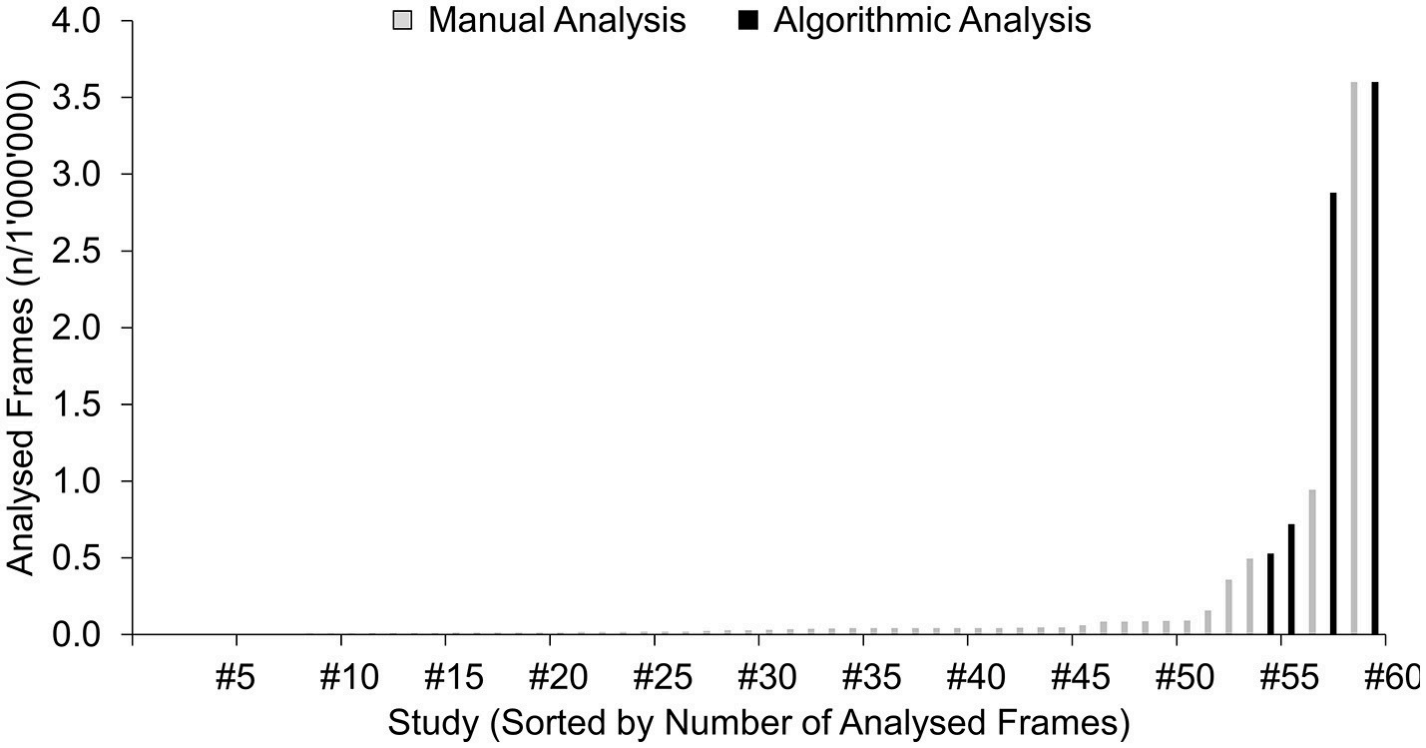
Estimated total numbers of analyzed frames in the 60 studies of the systematic review, sorted by quantity and distinguished by the pursued algorithmic (5 studies) vs. manual (55 studies) approach to gaze-cue allocation.

Beyond these advantages, stationary eye-trackers are particularly appealing with direct application of algorithmic allocation procedures, thus shedding the tedious, manually-conducted gaze-allocation process. Therefore, it should be no surprise that all studies that explicitly report a computerized, algorithmic gaze-cue allocation acquired gaze-data with a stationary eye-tracker (5/5; 100.0%). The remarkable benefit achieved by such an algorithmic approach to the gaze-cue allocation is illustrated in Figure 6, in which the studies included in the present review are sorted by total numbers of analyzed frames (roughly estimated from numbers of analyzed participants, trials per participant and eye-tracker frequencies, as specified in the respective papers and assuming the analysis of a 2-s interval per trial). Clear from the chart's bars on the right, five of the seven studies with the highest numbers of analyzed frames had pursued an algorithmic approach to gaze-cue allocation. For the remaining study with estimated total numbers of analyzed frames of 3.6 million (Piras et al., 2010), a semi-automatized approach was applied with algorithmic analyses of gaze behavior (i.e., detection of fixation and saccades) and, nevertheless, required a manual assigning of gaze-cue allocation (thus classifying these studies as “manual”). Comparatively, the resulting magnitude of gaze data available from all five studies pursuing an algorithmic approach (7.8 million analyzed frames) provides more data as the total estimated sum of 6.9 million analyzed frames from all the remaining 55 mobile eye-tracker studies included in this review. Thus, despite the discussed disadvantages of stationary eye-trackers, pursuing an algorithmic approach to gaze-cue allocation certainly seems valuable for future research on natural gaze behavior in sports.

## Discussion

This systematic review of 60 studies conducted over the past 40 years on the dynamics of natural gaze behavior in sports reveals a large variety of researched tasks, performed analyses and derived measures. Particularly substantiated by comparisons between sub-samples of the included studies, sports-related eye-tracking research seems to increasingly strive to achieve the following quality criteria: (a) realistic viewing conditions (i.e., data acquisition in the field or at least large-screen projections) and (b) naturalistic responses (i.e., real-world movements rather than button presses or verbal responses) to optimize the external validity of the experimental conditions, as well as (c) precise measurements (i.e., high sample rates and robustness against movement-induced artifacts), and (d) the capability to analyse large gaze-data sets to increase the reliability of the measurements (which has become possible with the application of algorithmic approaches). However, mainly due to limitations of current eye-tracking technology, achieving all of these objectives can hardly be achieved simultaneously. Researchers are thus still faced with a polarizing trade-off between external validity on the one hand and objectivity and reliability on the other hand. Consequently, the studies in the present review can be placed along a continuum based on the researchers' emphasis on either one or the other aspect of high-quality eye-tracking research on natural gaze behavior in sports.

On one end of this continuum, studies focus on the value in the measures, such as the study of Ryu et al. (2014) that examined the effects of different viewing conditions on gaze behavior and decision-making in complex basketball game-play situations. In this study, the use of a high-frequency, stationary eye-tracker (250 Hz), and a button-response system not only guaranteed high measurement accuracy, but also allowed for the online-blurring of either central or peripheral regions of the visual field for controlled experimental manipulation. However, the presentation of third-person videos on a computer screen, the movement-restricted sitting position of the participant as well as the unnatural motor response raises considerable doubts whether the findings can claim transferability to real-world situations (c.f. Mann et al., 2007; Dicks et al., 2010; Gegenfurtner et al., 2011).

On the other end of the continuum, studies can be identified with an aim to optimize external validity, as for instance, the study by Hüttermann et al. (2014) that investigated penalty takers' gaze, decision-making and shooting performance in a football-penalty task. By using a mobile eye-tracker in a real-world setting, the authors placed an emphasis on the external validity of the test conditions. However, by doing so, they had to relinquish some experimental control with respect to the variable behavior of the goalkeeper, the large workload for the manual analysis of 30 trials of 22 participants, and the reduced precision of the measurements due to potential slippage artifacts of the mobile device. Further, the resulting application of a rather low-frequency eye-tracker (30 Hz) makes the analysis of saccadic eye-movements, as conducted by the authors, debatable (cf. Andersson et al., 2010, for a recommendation of minimally 50 Hz and optimally 200 Hz for saccade detection).

In the majority of cases, however, the researchers faced the trade-off by compromising between the incompatible demands. In our view, the most promising approach in this respect was presented by Mann et al. (2013) (although this study, owing to the low number of AOIs, could not be included in the present review). To compare expert and near-expert cricket batters' gaze behaviors, the authors conducted a laboratory study in which the application of a ball-delivery machine allowed for the control of ball-flight specifics (direction, speed, and length). The visual stimuli were presented on a life-sized screen attached to the ball-delivery machine, which released a ball synchronized to the projected thrower's movement. In this way, experimental control was secured, as both groups of batters were confronted with exactly the same stimuli; while at the same time, the real-world situation was re-enacted as close as possible, with the batters instructed to hit the ball delivered by the realistic cue presentation as is required in a real cricket match. The only two flaws in this—otherwise perfectly planned—design seem to concern, first, the eye-tracking device that operated on a rather low frequency of 25 Hz and, second and not independent from the sample-rate issue, the manual approach to gaze-cue assignment that resulted in a rather small amount of analyzed gaze data.

As follows, the hereby illustrated compromise problem can be traced back to two—conceptually distinct—causes; the first concerning the state-of-the-art of eye-tracking technology and methods of gaze-data analysis, and the second relating to a desirable high degree of experimental control. Both of these causes deserve some closer inspection.

(1) The compromise is notably apparent when considering the analysis of saccadic (e.g., Ryu et al., 2014: 250 Hz eye-tracker) or micro-saccadic (e.g., Piras et al., 2015: 500 Hz eye-tracker) gaze behavior. The application of high-frequency mobile eye-trackers comes with the difficulty of handling very large sets of raw gaze data. For instance, with 200 frames per second for a number of seconds per trial, a number of trials per participant and a number of participants per sample, a manual approach to raw-data processing alone would be impossible. Therefore, efficient analysis methods need to be developed, such as those proposed by Kredel et al. (2015). In this setup, a mobile eye-tracker with a relatively high sample rate is integrated into a motion-capture system (e.g., Vicon). Therefore, participants are not restricted in their movements, enabling them to execute natural responses. Since the current position of the eye-tracker is recorded by the motion-capture system, a gaze vector in the laboratory frame of reference can be calculated, and thus giving the precise intersection of this vector with a projection screen, displaying videos of sports situations from a first-person perspective. By digitizing the videos to obtain the 2D coordinates of predefined AOIs in each video frame beforehand, algorithmic allocation of the gaze vector and hence, automatic processing of very large gaze-data sets becomes readily available. Not only does this setup dramatically economize the analysis of mobile eye-tracking data, but this algorithmic approach also brings multiple advantages; namely, (a) considerably increased objectivity in the gaze-cue-allocation process, (b) the opportunity to add AOIs to analysis that are related to more than one object (e.g., the space centrally between AOI1 and AOI2), (c) the possibility to include future or previous AOIs in the algorithmic processing that do not appear in the currently analyzed frame (e.g., the location where the ball will hit the ground), and (d) a synchronized capturing of the participant's movements, which in turn provides the opportunity to include action-related events in the algorithmic gaze analysis (e.g., the moment of response initiation).

In the course of attempting to improve this capability some further flaws of currently available eye-trackers should be tackled as well. These flaws particularly concern the occurrence of slippage artifacts due to head accelerations, inert masses distant to head rotation axes, and eye-tracker goggles insufficiently fixed to the head. In addition, an increased miniaturization of supply, storage or transmitting units is highly desirable as well as an improved wireless control architecture to further optimize the participants' mobility. Moreover, when aiming for field applications that require interactions with, for instance, opponents or balls, the risk of injuries must be minimized, most apparently by removing hazardous eye-tracker parts (i.e., cameras or mirrors) from locations in front of the eyes. Such hardware advancement would additionally and desirably increase the participant' field of view. And finally, for certain field conditions, such as in snow sports or in bright sunlight, considerable measures should be taken to confront specific issues of low temperatures, excessive light reflections, and the system's water resistance.

(2) Assuming that all the technological challenges sketched above will be successfully met over the next years, one particular issue regarding the design of sports-related eye-tracking research still remains: the issue of conducting studies under laboratory vs. field conditions. In this regard, at least conceptually, the integrated laboratory setup proposed by Kredel et al. (2015) could be successfully transferred to the field if the following two requirements were fulfilled: (a) the 3D location and orientation of the mobile eye-tracker is captured by an appropriate (local or global) positioning system (e.g., a high-accuracy differential GPS), and (b) the 3D locations of crucial AOIs are known in relation to the current eye-tracker position (e.g., by similar tracking devices attached to limbs of an opponent player).

However, even if these further requirements were achieved, it must be emphasized in the context of the present discussion that the fundamental gap between laboratory and field research, with the substantial trade-off between experimental control and real-world conditions, cannot be closed by technological means. More precisely, this difference cannot be resolved as long as virtual-reality laboratory setups that perfectly mimic the relevant real-world conditions are not available. In the case of virtual-reality, the demands of experimental control and of acting in a “real” environment could be simultaneously achieved. For the vast majority of sports situations, however, the realization of such high-level virtual environments should not be expected to be ready for use in the near future. Until this form of virtual-reality becomes a reality, scientists researching dynamic gaze behavior in sports will have to deal with the trade-off sketched above. The inevitable decision then to favor one or the other demand requires careful deliberation of the relative importance of (a) controlled laboratory conditions and (b) real-world field conditions. On which arguments this decision should be based and what respective consequences are incurred by certain biases will now further be discussed in detail.

Certainly, if (a) experimental control was crucial for the investigation of interest, this starting point forms a strong argument for preferring laboratory research. Quite obviously, for example, gaze-related effects of slight deviations of an opponent's backswing movement from his or her standard stroke can be reliably put to empirical test only by the controlled presentation of appropriate videos. In this case, however, though constrained by the laboratory environment to a certain degree, the experimenter should simultaneously strive for a maximization of the external validity of the test conditions. These efforts particularly refer to the visualization conditions (i.e., life-size and even multi-wall projections, preferably stereoscopic and with head-tracking for perspective changes), thereby increasing the degree of immersion and by this means, reducing transfer losses due to the accepted compromise on the congruence to the actual sports situation. Beyond, to further optimize the integration of real-world conditions in an experimental setup, natural(istic) motor responses of the participants should be favored over verbal or button-press responses, rather requiring sports-specific actions (as done, e.g., in the above-sketched cricket study by Mann et al., 2013) or at least letting the participants mimic the naturally performed movements (as, e.g., Vansteenkiste et al., 2014a).

If, however, (b) the empirical work first and foremost requires the investigation of gaze behavior in unrestricted real-world conditions, then it might be better to compromise the standardization of the data-acquisition setup, and thus the degree of experimental control. This alternative seems to be particularly preferable when investigating sports in which the gaze behavior is highly dependent on specifics of the natural environment. For instance, this would apply when trying to study gaze behavior in skydiving, or generally, sports that bring about perceptual sensations that, even in the far future, can hardly be satisfactorily re-enacted in a virtual-reality laboratory environment. However, as long as the available eye-tracking technology does not allow for the algorithmic analysis of gaze data gathered under respective environmental conditions, favoring field research will inevitably limit the reliability of the obtained empirical results.

In summary, as derived from the present review, researching dynamic gaze behavior in sports is most burdened by the trade-off between laboratory or field research, which can be reduced by further technological developments but—for principle reasons—will never be solved by this means alone. Nevertheless, in order to increase the amounts of data acquired for the derivation of reliable results, the development of high-frequent and robust eye-trackers, integrated in positioning systems to allow for the algorithmic gaze-cue allocation of large amounts of raw gaze data, stands as the major challenge of sport science. In our view, in order to raise current research efforts to a substantially higher level, this challenge deserves particular attention over the forthcoming decade.

## Author contributions

All authors listed have made a substantial, direct and intellectual contribution to the work, and approved it for publication.

### Conflict of interest statement

The authors declare that the research was conducted in the absence of any commercial or financial relationships that could be construed as a potential conflict of interest.
